# SARS-CoV-2 seroprevalence in pregnant women during the first three COVID-19 waves in The Gambia

**DOI:** 10.1016/j.ijid.2023.08.012

**Published:** 2023-08-15

**Authors:** Ramatoulie E. Janha, Alasana Bah, Hawanatu Jah, Fatima Touray, Yahaya Idris, Saikou Keita, Yassin Gaye, Samba Jallow, Tisbeh Faye-Joof, Baboucarr Njie, Rachel Craik, Nuredin I. Mohammed, Peter von Dadelszen, Umberto D’Alessandro, Anna Roca, the PRECISE Network, Laura A. Magee, Hiten Mistry, Marie-Laure Volvert, Thomas Mendy, Lucilla Poston, Jane Sandall, Rachel Tribe, Sophie Moore, Tatiana Salisbury, Marleen Temmerman, Angela Koech, Patricia Okiro, Geoffrey Omuse, Grace Mwashigadi, Consolata Juma, Isaac Mwaniki, Alex Mugo Joseph Mutunga, Moses Mukhanya, Onesmus Wanje, Marvin Ochieng, Emily Mwadime, Esperança Sevene, Corssino Tchavana, Salesio Macuacua, Anifa Vala, Helena Boene, Lazaro Quimice, Sonia Maculuve, Eusebio Macete, Inacio Mandomando, Carla Carillho, Donna Russell, Hannah Blencowe, Veronique Filippi, Joy Lawn, Matt Silver, Joseph Waiswa, Ursula Gazeley, Prestige Tatenda Makanga, Liberty Makacha, Reason Mlambo, Andrew M. Prentice, Melisa Martinez-Alvarez, Brahima Diallo, Abdul Sesay, Sambou Suso, Fatoumata Kongira, Modou F.S. Ndure, Lawrence Gibba, Abdoulie Bah, Yorro Bah, Alison Noble, Aris Papageorghiou, Judith Cartwright, Guy Whitley, Sanjeev Krishna, Marianne Vidler, Jing Larry Li, Jeff Bone, Mai-Lei Maggie Woo Kinshella, Domena Tu, Ash Sandhu, Kelly Pickerill, Ben Barratt

**Affiliations:** King’s College London; Aga Khan University, Nairobi; Centro de Investigação de Saúde de Manhiça; Donna Russell Consulting; London School of Hygiene and Tropical Medicine; Midlands State University; MRC Unit The Gambia at LSHTM; University of Oxford; St George’s, University of London; University of British Colombia; Imperial College London; 1 Medical Research Council Unit The Gambia at the London School of Hygiene and Tropical Medicine, Atlantic Boulevard, Banjul, The Gambia; 2 Department of Women and Children’s Health, School of Life Course Science, Faculty of Life Sciences and Medicine, King’s College London, London, UK; 3 Nuffield Department of Women’s and Reproductive Health, University of Oxford, Oxford, UK

**Keywords:** Antibodies, COVID-19, Pregnancy, Seroprevalence, sub-Saharan Africa, The Gambia

## Abstract

**Objectives:**

SARS-CoV-2 transmission in sub-Saharan Africa has probably been underestimated. Population-based seroprevalence studies are needed to determine the extent of transmission in the continent.

**Methods:**

Blood samples from a cohort of Gambian pregnant women were tested for SARS-CoV-2 total receptor binding domain (RBD) immunoglobulin (Ig) M/IgG before (*Pre-pandemic:* October-December 2019) and during the pandemic (*Pre-wave 1:* February-June 2020; *Post-wave 1*: October-December 2020, *Post-wave 2*: May-June 2021; and *Post-wave 3*: October-December 2021). Samples reactive for SARS-CoV-2 total RBD IgM/IgG were tested in specific S1- and nucleocapsid (NCP) IgG assays.

**Results:**

SARS-CoV-2 total RBD IgM/IgG seroprevalence was 0.9% 95% confidence interval (0.2, 4.9) in *Pre-pandemic*; 4.1% (1.4, 11.4) in *Pre-wave 1*; 31.1% (25.2, 37.7) in *Post-wave 1*; 62.5% (55.8, 68.8) in *Post-*wave *2* and 90.0% (85.1, 93.5) in *Post-wave 3*. S-protein IgG and NCP-protein IgG seroprevalence also increased at each *Post-wave* period. Although S-protein IgG and NCP-protein IgG seroprevalence was similar at *Post-wave 1*, S-protein IgG seroprevalence was higher at *Post-wave 2* and *Post-wave 3*, (prevalence difference 13.5 [0.1, 26.8] and prevalence ratio 1.5 [1.0, 2.3] in *Post-wave 2*; and 22.9 [9.2, 36.6] and 1.4 [1.1, 1.8] in *Post-wave 3* respectively, *P* <0.001).

**Conclusion:**

SARS-CoV-2 transmission in The Gambia during the first 3 COVID-19 waves was high, differing significantly from official numbers of COVID-19 cases reported. Our findings are important for policy makers in managing the near-endemic COVID-19.

## Introduction

SARS-CoV-2, the causative agent of the COVID-19, was first identified in December 2019 in Wuhan, China [1]. SARS-CoV-2 was rapidly transmitted globally, disrupting structures and systems, and causing millions of deaths. Globally, there were 278.7. million COVID-19 cases and 5.39 million attributable deaths by December 2021 [2], 21 months after the World Health Organization (WHO) declared COVID-19 a pandemic [3]. Nigeria reported the first case in sub-Saharan Africa (SSA) on 28 January 2020 [4]. The sub-Saharan Africa (SSA) continent had the worst epidemiological modelling predictions of cases, hospitalizations and deaths [5]. SARS-CoV-2 infection during pregnancy is of particular concern, as it increases the rates of adverse maternal and neonatal outcomes [6]. Nevertheless, the impact of COVID-19 in SSA was lower than expected, with 7.1 million cases and 155,300 deaths by December 2021, i.e., 2.5% of all cases and 2.9% of all deaths worldwide, despite Africa representing 17.4% of the global population [2].

Some underpinning factors in Africa, specifically in SSA, may explain the discrepancy between predictions and actual burden. One of these is the limited capacity for diagnosis, surveillance and testing for SARS-CoV-2, exacerbated by lack of systematic death registrations in most SSA countries. Moreover, older age is a major risk factor for severe COVID-19 and associated deaths. As the SSA population is young, i.e., in 2022, the median age was 18.8 years, with 40% aged 14 years and younger, this may partially explain the low number of reported deaths [7]. The communities’ perceptions of COVID-19 and avoidance of healthcare facilities [8] during the pandemic may have added to underdiagnosis of cases and non-certification of COVID-19 as the cause of death. However, there is emerging evidence that transmission in SSA has been at least as high or even higher than in other continents [9], although these data should be interpreted with caution as some studies have important limitations. Most of them do not represent the entire population, rather specific groups, generally high-risk groups [10]. In addition, SARS-CoV-2 prevalence may be overestimated as SARS-CoV-2 antibodies may cross-react with other viral or parasitic antibodies in the region, such as IgG antibodies against the spike and nucleocapsid proteins of different human coronaviruses: HCoV-OC43, HKU-1, NL63 and 229E, as well as SARS and MERS [11], and IgG directed against *Plasmodium falciparum* antigens where malaria is endemic [12]. To overcome such challenges, the WHO recommends (i) the use of multiple assays to confirm seropositivity for SARS-CoV-2, including testing positive or equivocal samples with neutralizing assays; (ii) repeated cross-sectional surveys to monitor the population serological profile [13]; and (iii) testing assay specificity [14], as the multiple available assays consist of a combination of serological tests targeting separate groups or monomers of specific antigens.

The Gambia is a small country situated in West Africa with a population of 2.57 million [15]. The objective of this study was to describe the seroprevalence of SARS-CoV-2 before (as a measure of specificity) and following each of the first 3 waves among pregnant women living in peri-urban and rural Gambia who participated in the PRECISE study [16]. In general, pregnant women represent a healthy young adult population and seroprevalence in this group should represent seroprevalence in adult healthy population.

## Methods

### COVID-19 and the national response in The Gambia

**Figure 1:**
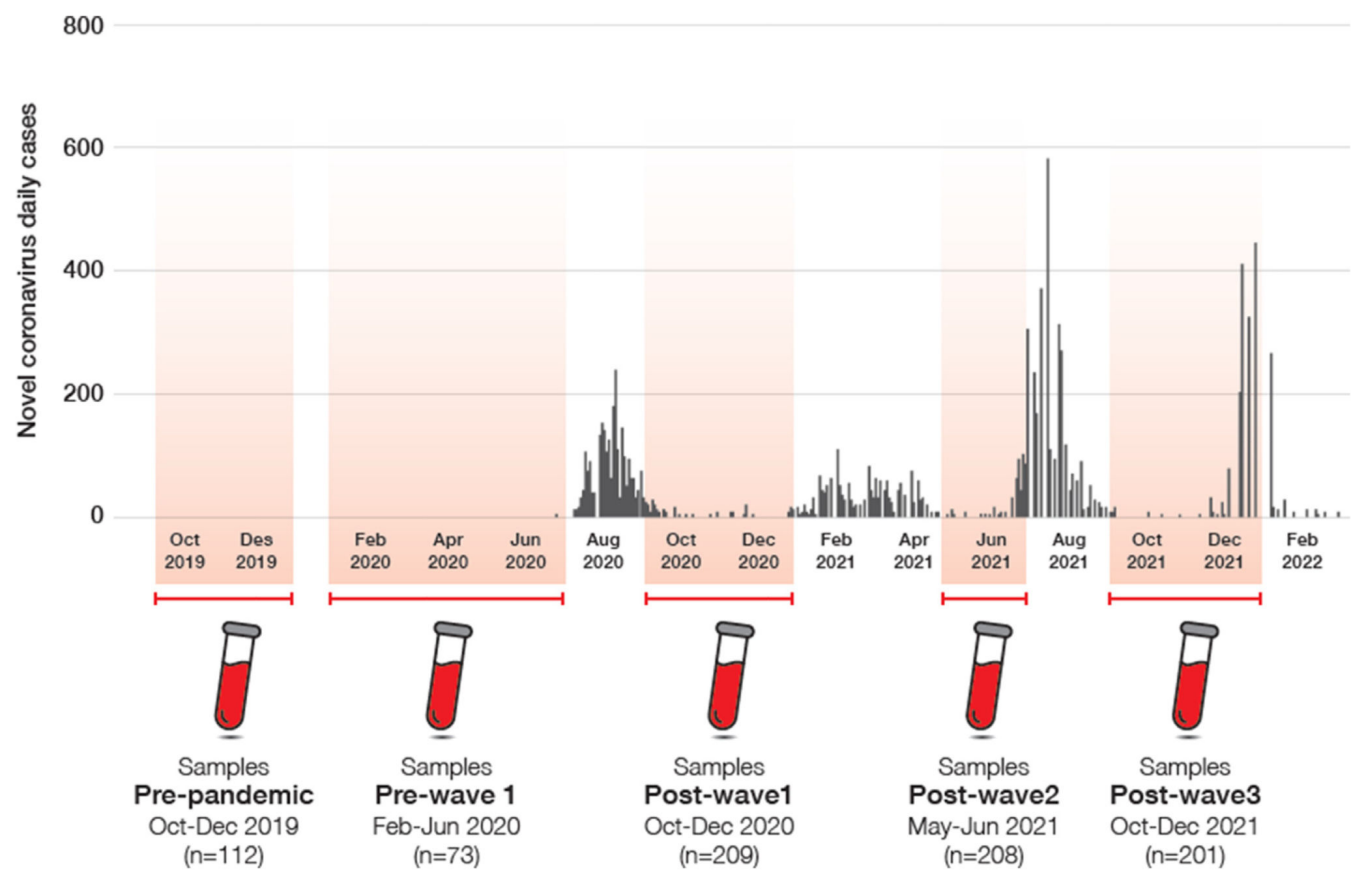
SARS-CoV-2 transmission waves embedding study design. A schematic design showing sampling time periods and sample-sizes per time period. The shadowed areas are the period of sample collection. Samples were grouped into *Pre-pandemic, Pre-wave 1* and three pandemic time periods linked to the first three COVID-19 waves as *Post-wave* time periods. *Source*: https://www.moh.gov.gm/wp-content/uploads/2022/09/GMB-COVID-19-Situational-Report-452_2022_10th_24th_September_2022.pdf.

The Gambia’s first official COVID-19 case was reported on 16 March 2020 [17]. By December 2021, The Gambia had registered 3 SARS-CoV-2 transmission waves dated as follows: first, July-September 2020; second, January 2021; and third, July 2021 (Figure 1). The first and third waves were more intense, with higher peaks of official daily cases [18] with the delta variant being the most frequent during the third wave [19]. The second wave concurred with the global transmission of the delta/B.1.617.2 sub-type variant, described in The Gambia at the time [19].

Following the diagnosis of the first few official cases, a state of emergency was declared [20]. Briefly, authorities prompted stringent response measures, including border closures and a national lockdown (schools, worshipping grounds, and non-essential business activities), initially for 3 months. Mandatory wearing of face masks and observing physical distancing in public places were enforced [20]. Epidemiological surveillance consisted of isolation, quarantine and reverse transcription-polymerase chain reaction testing of suspected cases and travelers and contacts-tracing of confirmed cases. Surveillance was however mostly carried out in the urban Western Region. Vaccination coverage was very low; by December 2021, only 10.2% of the total population (and <1% in our study area population) was fully vaccinated with 2 doses of Astrazeneca/Sinopharm or 1 dose of Johnson and Johnson [21].

### Study design – The Pregnancy Care Integrating Translational Science, Everywhere study

This is an ancillary study of the PRECISE study (Pregnancy Care Integrating Translational Science, Everywhere) [16], in which a pregnancy cohort was recruited in The Gambia, Kenya, and Mozambique, with the collection of extensive sociological and clinical data and biological samples throughout woman’s pregnancy, until 6 weeks post-delivery [16].

### Pregnancy Care Integrating Translational Science, Everywhere The Gambia

In The Gambia, the study was implemented in Farafenni, North Bank Region, 116 km from the capital city Banjul. Farafenni is divided into rural and urban areas and forms an important commercial border with Senegal. Study participants were pregnant women attending antenatal care at three health facilities: Maternal Newborn Child and Adolescent Health Clinic in Farafenni (urban), Illiasa and Ngayen Sanjal (rural) health centers. Farafenni General Hospital is the main referral hospital for the North Bank Region, population 225,516 [22], and Illiasa and Ngayen Sanjal are 17 km west and 20 km east of Farafenni, respectively.

The Gambian PRECISE cohort includes 1253 pregnant women aged 16-49 years. Participants were recruited at their first antenatal visit which could be at any time during their pregnancy (mainly in the second trimester). A second antenatal visit was scheduled where possible between 28 weeks’ gestation and the onset of labor (in the third trimester), and at least 4 weeks after their booking visit. Women were then seen during their admission for giving birth if that occurred in a participating healthcare center. The last study visits occurred 6 weeks to 6 months after delivery. At each study visit, blood samples were collected (between October 2019 and March 2022). As stated, demographic, epidemiological, and clinical information was collected during study visits. In our study population, vaccination status was not collected.

### SARS-CoV-2 seroprevalence study

For the SARS-CoV-2 seroprevalence study presented here, we considered five sera collection time periods that corresponded to the SARS-CoV-2 waves observed in The Gambia [19]: (i) *Pre-pandemic* (October - December 2019) before any case was detected in The Gambia; (ii) *Pre-wave 1* (February - June 2020) during which few cases were diagnosed but no wave had yet started and the stringent 3-months national lockdown implemented; (iii) *Post-wave 1* (October - December 2020); *Post-wave 2* (May - June 2021); and (v) *Post-wave 3* (October - December 2021) (Figure 1). The *Pre-pandemic* period was included as an additional step to determine specificity of the test used to measure the study primary endpoint in our population as no SARS-CoV-2 transmission in The Gambia during these months is expected.

For each time period, only one sample per woman was included although samples from the same woman could have been selected for other periods.

### Study endpoints

The primary endpoint was the SARS-CoV-2 seroprevalence (immunoglobulin [Ig] M/IgG against SARS-CoV-2 receptor binding domain [RBD] proteins) at each time-period as determined by the WANTAI. Secondary endpoints include IgG seroprevalence to specific SARS-CoV-2 proteins (spike S1-subunit IgG and nucleocapsid protein, NCP-IgG) (EUROIMMUN test).

### Serum collection and preparation

Venous blood (6 ml) was collected into serum separation tubes (BD Vacutainer SST™ tubes, Fisher Scientific), allowed to clot, and centrifuged at 2000 g for 15 minutes. Separated sera were kept at −80°C until serological analyses.

### Lab analysis and interpretation

(a) WANTAI immunoassay. Sera were tested using the WANTAI SARS-CoV-2 antibody enzyme-linked immunosorbent assay (ELISA) Rapid Diagnostic Test kit (Wantai, Beijing, China) for qualitative detection of SARS-CoV-2 total RBD IgM/IgG [23]. The WANTAI immunoassay microwell strips are pre-coated with recombinant RBD of the spike protein antigen incorporated into a lateral flow ELISA design. The assay has a manufacturer-validated sensitivity of 98.7% and specificity of 98.6% in participants from China [23]. An independent clinical agreement validation of the WANTAI assay was conducted at the Frederick National Laboratory for Cancer Research in the United States in July 2020 [23]. Each microwell plate had a blank calibrator, 50 μl control, 100 μl of serum. Following two-steps of incubation at 37°C, washing, horse radish peroxidase-conjugation and colorization, absorbance was read at 450 nm. After blanking and cut-off determination, samples with absorbance/cut-off ratio ≥1 were positive for WANTAI SARS-CoV-2 total RBD IgM/IgG.
(b) EUROIMMUN immunoassay. Two EUROIMMUN anti-SARS-CoV-2 ELISA test kits (EUROIMMUN, US, Germany) [24,25] were further performed on all WANTAI tested-positive samples (Supplementary Figure 1). The EUROIMMUN microplates are pre-coated separately with recombinant S1 domain of spike protein, for detecting IgG antibodies against the S1-subunit; and modified NCP for detecting IgG against NCP. The S1-protein IgG test has a sensitivity of 80% and a specificity of 98.5% [24]; and the NCP-protein IgG test has a sensitivity of 94.6% and a specificity of 99.8% on European patients’ sera [25]. Samples were diluted in sample buffer. Each well had 100 μl of calibrators. Plates were incubated, washed and enzyme-conjugated, followed by absorbance measurement at 450 nm. Results for controls and samples were calculated considering the extinction of the calibrator. The results were interpreted as: ratio<0.8: negative; ratio ≤0.8 to <1.1: borderline/indeterminate; and ratio ≤1.1: positive.

### Statistical analysis

Categorical variables, including SARS-CoV-2 seroprevalence, were summarized using proportions. Chi-square or Fisher’s exact test were used where appropriate to test for differences in proportions between time periods. Continuous variables were summarized using median (interquartile range).

SARS-CoV-2 seroprevalence by WANTAI was expressed as the proportion (95% confidence interval [CI]) of WANTAI-positives to the total number of tested samples per time period. SARS-CoV-2 seroprevalence by EUROIMMUN was expressed as the proportion (95% CI) of EUROIMMUN-positives to the total number of samples tested per time period. EUROIMMUN borderline results were considered as negatives.

Prevalence ratios (PR) and prevalence differences (PD) between EUROIMMUN S-protein IgG and NCP-protein IgG antibodies at each wave were calculated using generalized linear models (glm) with robust standard errors. We used Bonferroni method throughout to account for multiple comparisons (due to multiple time periods).

To determine the proportions of new infections at each post-wave period (considering any seroprevalence by WANTAI), our assumptions were that after one infection women remained seropositive throughout the study and women tested at each period represented a random sample from the PRECISE cohort. Therefore, we calculated the percentage of new infections as the increased prevalence in the population at risk (percentage of population uninfected in the preceding wave). All data management and analyses for this study were performed using Stata version 17.0 [26].

## Results

### Participants’ baseline characteristics

803 women were included in the five sera collection time periods, with 112 in *Pre-pandemic*, 73 in *Pre-wave 1*, 209 in *Post-wave 1*, 208 in *Post-wave 2* and 201 in *Post-wave 3*. Baseline characteristics of women were similar at the different time points, with a median age of 26 years, both Mandinkas and Wolofs being the most common ethnic groups, median household size of 9 individuals per household and median parity 2-4 children per woman (Supplementary Table 1).

### SARS-CoV-2 total receptor binding domain seroprevalence (WANTAI)

**Figure 2:**
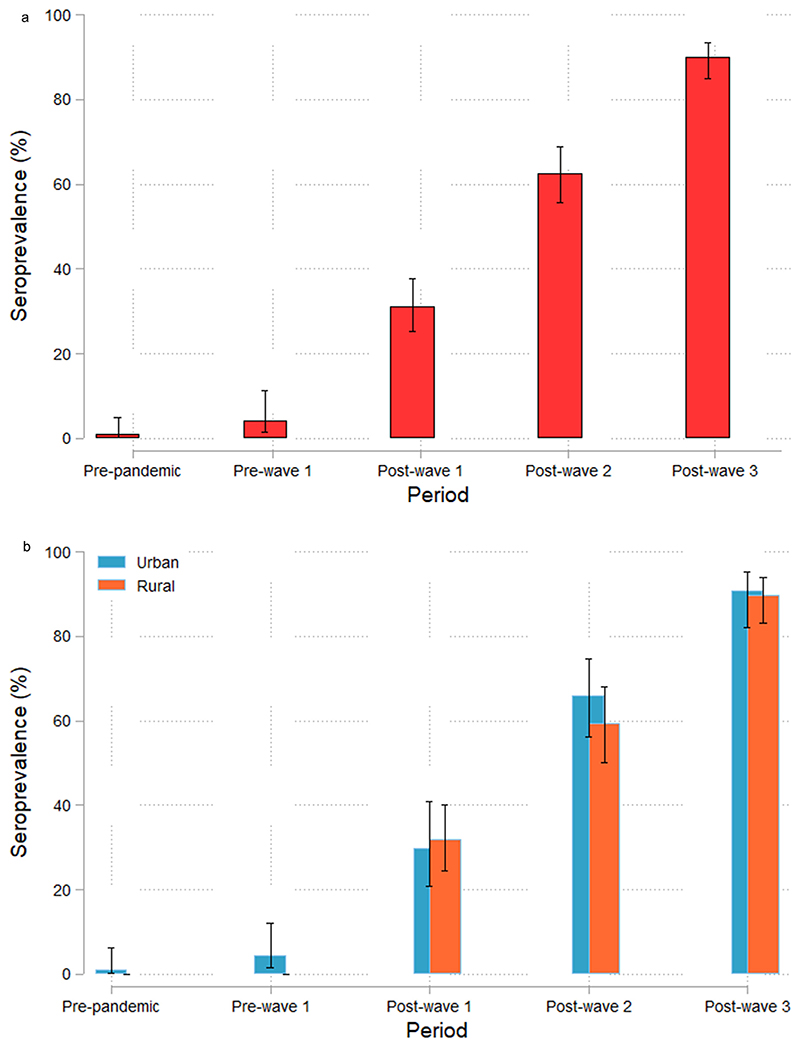
SARS-CoV-2 seroprevalence at each study period: (a) Overall; (b) Stratified by rural and urban health facilities (WANTAI test). Chart showing SARS-CoV-2 total RBD immunoglobulin G/immunoglobulin M antibodies’ seroprevalence with 95% confidence interval (a) per time period and (b) for rural and urban Farafenni in the *Post-wave* time periods.

Overall, SARS-CoV-2 seroprevalence was low in the *Pre-pandemic period* at 0.9% 95% CI (0.2, 4.9) and the *Pre-wave 1 period* at 4.1% (1.4, 11.4) (Table 1, Figure 2a). SARS-CoV-2 seroprevalence significantly increased over time, from 31.1% (25.2, 37.7) in the *Post-wave 1 period* to 90.0% (85.1, 93.5) in the *Post-wave 3 period* (*P* <0.001, Figure 2a, Table 1 and Table 2). The stratified analysis of seroprevalence shows similar results for urban and rural Farafenni (Table 1, Figure 2b).

### SARS-CoV-2 protein specific seroprevalence: spike (S1-subunit protein) specific immunoglobulin G and nucleocapsid - nucleocapsid protein specific immunoglobulin G

**Figure 3:**
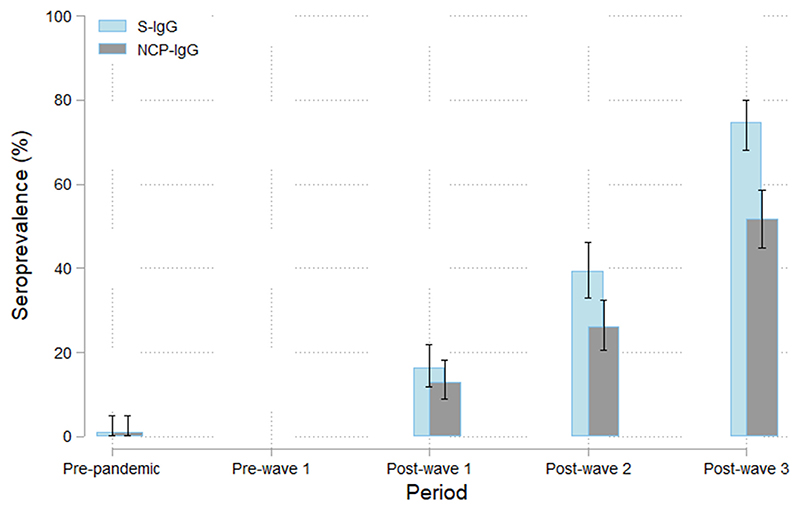
Prevalence of SARS-CoV-2 IgG sero-positivity against S-specific protein IgG and NCP-specific protein IgG. Chart showing SARS-CoV-2 spike S1-subunit protein specific (S-IgG [blue]) and (NCP-IgG [grey]) antibodies’ prevalence and 95% confidence interval per time period. Ig, immunoglobulin; NCP, nucleocapsid protein specific; S, spike.

S-protein specific SARS-CoV-2 seroprevalence increased significantly in the post-wave periods, from 16.3% (11.9, 21.9) in the *Post-wave 1 period* to 74.6% (68.2, 80.1) in *the Post-wave 3 period*. Increase of seroprevalence between *Post-wave 1, -2* and *-3* was significant for all comparisons, in terms of PR and PD (Supplementary Table 2, Figure 3).

Similarly, NCP-protein specific SARS-CoV-2 seroprevalence increased significantly in the *post-wave* periods, from 12.9% (9.0, 18.1) in the *Post-wave 1 period* to 51.7% (44.9, 58.6) in the *Post-wave 3 period*, with significant increase for each comparison, in terms of PR and PD (Supplementary Table 2, Figure 3).

SARS-CoV-2 protein specific seroprevalence was similar for S-protein IgG and NCP-protein IgG in the *Post-wave 1 period* (*P* = 1.000 for PR and PD). However, SARS-CoV-2 protein specific seroprevalence was higher for S-protein IgG than NCP-protein IgG in the *Post-wave 2 period* PR 1.5 95% CI (1, 2.3), *P* = 0.061, PD 13.5 95% CI (0.1, 26.8), *P* = 0.047 and the *Post-wave 3 period* PR 1.4 95% CI (1.1, 1.8), *P* <0.001, PD 22.9 95% CI (9.2, 36.6), *P* <0.001, showing that the difference between S-protein IgG and N-protein IgG increases after the *Post-wave 1 period* (Supplementary Table 2).

### New infections by post-wave period

**Figure 4:**
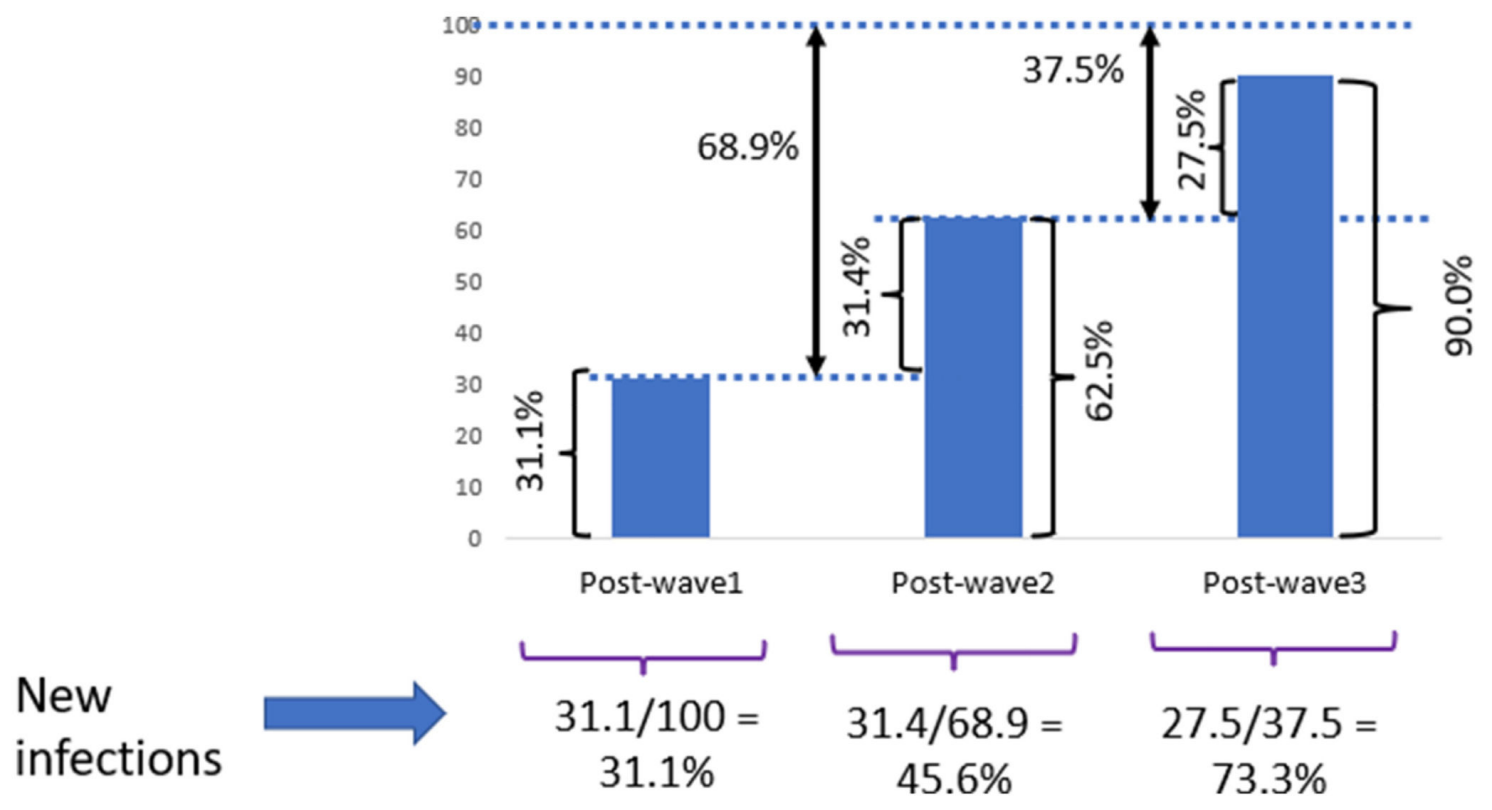
New infections in percentages at each of the *Post-wave* periods. Chart showing percentage of new infections in seronegative individuals (and re-infections) by anti- receptor binding domain (WANTAI) test at each *Post-wave* time period.

During *Post-wave 2*, 45.6% of the women at risk were newly infected. This percentage was up to 73.3% in the *Post-wave 3* period (Figure 4).

## Discussion

SARS-CoV-2 seroprevalence increased significantly among pregnant women living in Farafenni; between October 2019, before the first case of COVID-19 was diagnosed in The Gambia, and December 2021, after 3 epidemic waves. Most women acquired antibodies against SARS-CoV-2, indicating very high transmission in this area, and probably across The Gambia. The discrepancy between such high seroprevalence and the relatively modest number of reported COVID-19 cases is remarkable.

The delayed start of the epidemic in The Gambia was probably due to the measures taken by the Ministry of Health by declaring the state of emergency in March 2020, soon after the first SARS-CoV-2 case was diagnosed in The Gambia [20]. However, the stringent non-pharmaceutical interventions lasted only until June 2020, just before the first wave. Therefore, it is not surprising that in October-December 2020, after the first wave, about a third of pregnant women had SARS-CoV-2 antibodies as adherence to any non-pharmaceutical interventions (e.g., physical distancing, wearing of facemasks, decreasing mass gatherings at public places) from the moment the state of emergency was relaxed and the borders opened was low [17]. These findings confirm that SARS-CoV-2 transmission in The Gambia was much higher than those reported officially about COVID-19 cases and deaths. Indeed, a study analyzing the incidence of SARS-CoV-2 after the first wave among staff with standard risk of infection (excluding those working with infected patients) working at the MRC Unit The Gambia indicated that transmission was 80-fold higher than officially reported [20]. Interestingly, the high transmission of SARS-CoV-2 during the first wave did not translate in overall excess mortality, confirming that the epidemic has been milder than in other regions. Nevertheless, in Farafenni, it was detected an excess mortality among the vulnerable 65+ years age-group [27]. Transmission became even easier for the virus in the second and third waves when nationwide restrictions were eased further [17]. The third wave in The Gambia was dominated by the highly transmissible delta variant [19]. This is shown by the estimated 73.3% of the population at risk (seronegative individuals) infected during this wave, increasing the overall SARS-CoV-2 seroprevalence to 90%. We assume that sero-positive individuals were also infected during this wave. However, *Post-wave 3* also slightly encroached into the fourth wave, driven by the omicron variant [19]. Given the high infectivity rate of omicron, this variant may have also contributed to the increased seroprevalence in *Post-wave 3*.

The high SARS-CoV-2 total RBD antibodies’ prevalence described in our study is comparable to that reported from other West African countries at similar times. In Senegal, national SARS-CoV-2 seroprevalence was 28.1% in October-November 2020, at the end of their first transmission wave [28], similar to the prevalence in Farafenni at the same time. Furthermore, in Togo, nationwide seroprevalence in May-June 2021 was 64.3%, which is similar to the prevalence in Farafenni after the second wave [29].

The WHO-procured WANTAI test for standardization is widely used in Africa [9]. We measured the specificity of the test in our study population by analyzing samples collected before the detection of any COVID-19 case outside Wuhan (our *Pre-pandemic* period covers the period October to December 2019), and were able to confirm the high specificity of this test in our population as only 1 out of 112 samples was positive (likely, a false positive) in the *Pre-pandemic period*. A major strength of this study is that pre- and post-epidemic samples were collected from the same population and, therefore, were comparable, and provides confidence that seroprevalence was estimated correctly. This is important as other studies carried out in SSA suggested that the high prevalence of SARS-CoV-2 antibodies in some countries were due to the high cross-reactivity of samples and thus the low specificity of the tests used [11]. Four ELISA tests had lower specificity when used on African samples than European or American samples [30]. These tests included the two EUROIMMUN tests used in this study but not the WANTAI test. We did not determine the specificity of the EUROIMMUN assay as only samples positive by WANTAI were sub-sequently tested using the SARS-CoV-2 protein specific IgG tests (S-protein and NCP-protein). We also included in our analysis a time period after the first cases had been detected in The Gambia, but before the first wave of the pandemic was officially declared (*Pre-wave 1*). The seroprevalence in this period did not differ significantly from the *Pre-pandemic period*, indicating that transmission was probably low before the first wave was detected even in Farafenni, where the first wave may have started due to the porous border with Senegal [19].

The WANTAI test has a high specificity using *Pre-pandemic* sera in our population which strengthens our *Post-wave* results. On the other hand, the lower prevalence observed against S-protein IgGs and NCP-protein IgGs compared with SARS-CoV-2 total RBD IgM/IgG is expected as the latter includes both IgM and IgG antibodies making it more sensitive than both EUROIMMUN tests [23–25]. Hence, the EUROIMMUN tests are likely to underestimate sero-prevalence as these would not include recent infections. Notwithstanding, the IgG prevalence against the S-protein and NCP-protein was high and increasing over time with the occurrence of the COVID-19 waves. Interestingly, the prevalence of NCP-protein IgG is significantly lower than that of the S-protein IgG in the *Post-wave 2* period and *Post-wave 3 period*, but not in the *Post-wave 1 period*. These results suggest antibodies against S-protein are lasting longer than those against the NCP-protein [31], as the latter wane sooner while the former accumulates. Indeed, the first exposure to SARS-CoV-2 of women whose samples were collected in October - December 2020 was during the first wave and thus they had similar levels of S- and NCP-protein specific IgGs. However, during subsequent waves, some women were probably re-infected while others were infected for the first time and thus S-protein specific IgG had a higher prevalence than NCP-protein specific IgG after the second and third waves. An analysis of samples of approximately 39,000 individuals in a clinical laboratory in the United States to monitor SARS-CoV-2 S-protein and NCP-protein durability reveals that both protein-specific IgGs reached a peak of 90% positivity at 21 days post-index [32]. However, by 10 months the NCP-protein IgG seropositivity declined to 68.2%, whilst the S-protein IgG remained at a rate of 87.8%. The data describing longitudinal monitoring of SARS-CoV-2 humoral antibody responses mostly come from high-income countries. Our observation of a shorter durability of NCP-protein IgG than S-protein IgG is in line with those observations.

We need to acknowledge some limitations of our study. The surveys were conducted in a homogeneous population, in a specific region and a relatively narrow age group. Therefore, it is not possible to extrapolate these results to other age groups or regions. However, pregnant women are generally a healthy population who routinely visit hospitals for antenatal care and institutional delivery [33] and, thus, are often used as a proxy group for the whole population. In general, they continue doing similar activities than before pregnancy. Prevalence in rural and urban Farafenni was very similar, possibly suggesting such prevalence may be consistent across the country. In addition, the differences between S-protein specific IgG and NCP-protein specific IgG seroprevalence in *Post-waves -2* and *-3* may be confounded by vaccination as vaccination only generates antibody responses towards the S-protein. Although vaccination status was not available for study women, it is worth noting that vaccine coverage in the study area was less than 1% by December 2021 [21].

In conclusion, SARS-CoV-2 transmission in The Gambia was high during the first three COVID-19 waves. The high seroprevalence observed in our study combined with the former data of no excess mortality in The Gambia in 2020 [27] confirms that the clinical severity of infection during the COVID-19 epidemic has been milder in The Gambia than in other countries. Our results are important for policy makers to take decisions on how to manage the epidemic virus that is progressing into an endemic virus. Our study confirms that in settings with limited laboratory capacity, sero-prevalence studies are a powerful tool to understand the real burden of SARS-CoV-2 transmission.

## Supplementary Material

Supplementary material associated with this article can be found, in the online version, at 10.1016/j.ijid.2023.08.012.

figure 1

table 1

table 2

## Figures and Tables

**Table 1:** Overall SARS-CoV-2 seroprevalence (IgM and IgG combined) at each study period.

Area	Period	Positive (Total) n (N)	Prevalence (95% CI)
Overall	Pre-pandemic	1 (112)	0.9 (0.2, 4.9)
	Pre-wave 1	3 (73)	4.1 (1.4, 11.4)
	Post-Wave 1	65 (209)	31.1 (25.2, 37.7)
	Post-Wave 2	130 (208)	62.5 (55.8, 68.8)
	Post-Wave 3	181 (201)	90 (85.1, 93.5)
Urban	Pre-pandemic	1 (87)	1.1 (0.2, 6.2)
	Pre-wave 1	3 (68)	4.4 (1.5, 12.2)
	Post-Wave 1	23 (77)	29.9 (20.8, 40.8)
	Post-Wave 2	64 (97)	66 (56.1, 74.6)
	Post-Wave 3	68 (75)	90.7 (82, 95.4)
Rural	Pre-pandemic	0 (25)	0
	Pre-wave 1	0 (5)	0
	Post-Wave 1	42 (132)	31.8 (24.5, 40.2)
	Post-Wave 2	66 (111)	59.5 (50.2, 68.1)
	Post-Wave 3	113 (126)	89.7 (83.1, 93.9)

**Table 2:** Comparison of SARS-CoV-2 seroprevalence (IgM and IgG combined) between post-wave time-periods.

Name	Period	Estimate (95% CI)	*P*-value
Prevalence ratio			
WANTAI	PostWave2 vs PostWave1	2 (1.5, 2.7)	<0.001
SARS-CoV-2	PostWave3 vs PostWave1	2.9 (2.2, 3.7)	<0.001
	PostWave3 vs PostWave2	1.4 (1.3, 1.7)	<0.001
Prevalence difference			
WANTAI	PostWave2 vs PostWave1	31.4 (20.3, 42.5)	<0.001
SARS-CoV-2	PostWave3 vs PostWave1	58.9 (49.8, 68.1)	<0.001
	PostWave3 vs PostWave2	27.5 (18, 37.1)	<0.001

CI, confidence interval; Ig, immunoglobulin.
