# Supplementary figures and images for: SARS-CoV-2 seroprevalence in pregnant women during the first three COVID-19 waves in The Gambia
Source: Int J Infect Dis. Author manuscript; Available in PMC 2025 Sep 27. (PMC7618177; doi:10.1016/j.ijid.2023.08.012)

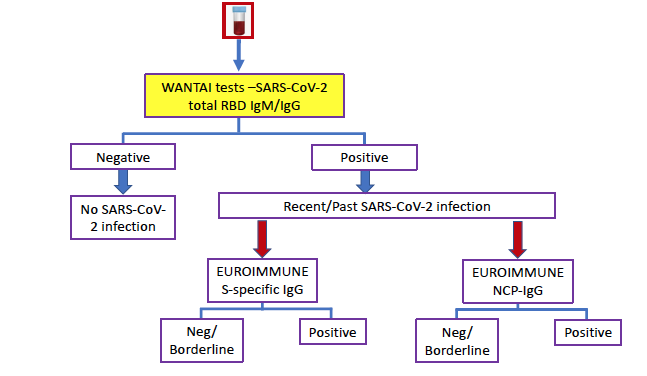


**Supplementary Figure 1**

Supplement: figure 1 [file EMS208245-supplement-figure_1.docx]
